# Comparison of Intracellular Stress Response of NCI-H526 Small Cell Lung Cancer (SCLC) Cells to Platinum(II) Cisplatin and Platinum(IV) Oxoplatin

**DOI:** 10.3390/cancers6031487

**Published:** 2014-07-08

**Authors:** Gerhard Hamilton

**Affiliations:** Ludwig Boltzmann Cluster of Translational Oncology, A-1090 Vienna, Austria; E-Mail: gerhard.hamilton@toc.lbg.ac.at; Tel./Fax: +43-1-40400-66270

**Keywords:** small cell lung cancer, cisplatin, oxoplatin, platinum(IV), phosphorylation, western blot, array, signal transduction, stress response, drug resistance

## Abstract

In attempts to develop an orally applicable platinum-based drug, platinum(IV) drugs which exhibit higher *in vivo* stability compared to the platinum(II) drug cisplatin were formulated. The first such chemotherapeutic agent, namely satraplatin, failed to receive approval. In the present work, we checked the initial cellular stress response of the chemosensitive NCI-H526 small cell lung cancer (SCLC) cells by determination of the relative phosphorylation of 46 specific phosphorylation sites of 38 selected proteins in a six hours response to cisplatin (platinum(II)) or oxoplatin (platinum(IV)), respectively. Oxoplatin is considered as prodrug of cisplatin, although several findings point to differences in intracellular effects. Cisplatin induced hyperphosphorylation of p38α MAPK and AMPKα1, whereas oxoplatin treatment resulted in increased phosphorylation of a large number of signaling proteins involved in stress response/drug resistance, including JNK, GSK-3α, AMPKα1, src kinases, STATs, CHK-2 and especially focal adhesion kinase (FAK). Cisplatin exerts markedly higher cytotoxicity upon four hours short-term exposure in comparison to oxoplatin and, correspondingly, the extended initial stress response to the platinum(IV) drug oxoplatin thus is expected to increase clinical drug resistance. Induction of a substantial stress response to any prodrug of a platinum-based compound may likewise limit the effectivity of its active metabolite(s), such contributing to the failure of selected derivatized platinum complexes.

## 1. Introduction

**Figure 1:**
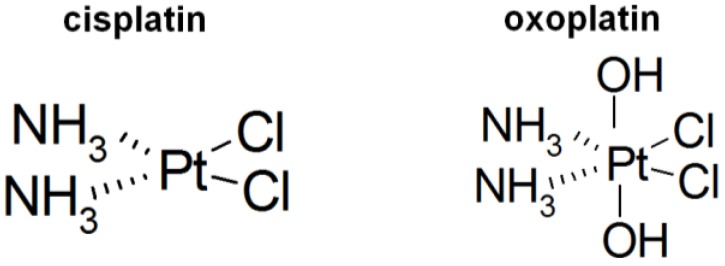
Structural formulas of cisplatin and oxoplatin.

Lung cancer is the leading cause of death worldwide and its incidence is increasing in women [1]. Approximately 15% of patients have small cell lung cancer (SCLC) and undergo treatment with a platinum-based regimen, comprising cisplatin/carboplatin along with a second chemotherapeutic drug, such as etoposide, docetaxel, gemcitabine, pemetrexed, or vinorelbine [2,3]. Beside SCLC, cisplatin (*cis*-diaminodichlorido-platinum(II), Figure 1) was established as an active agent against diverse tumor types including testicular, ovarian, head and neck, bladder, and esophageal cancer [4,5]. However, administration of cisplatin is restricted by serious side effects such as nephrotoxicity, emetogenesis, neurotoxicity, and the appearance of inherent and acquired resistance to the drug, leading to a dismal prognosis [5,6]. Additionally, cisplatin has to be applied intravenously whereas development of orally applicable platinum-based drugs would allow for outpatient care. The first oral platinum coordination complexes, namely picoplatin (platinum(II)) and satraplatin, failed to gain approval for clinical use [7,8]. Molecules containing a platinum(IV) central atom have several desirable properties over platinum(II) drugs, such as chemical inertness and reduced reactivity, leading to long half-lives in the circulation, and lower toxic side effects and cytotoxicity in cisplatin-resistant tumor cell lines [9]. Distinct pharmacokinetic characteristics of these platinum(IV) complexes are determined by different axial ligands. A platinum(IV) compound, namely oxoplatin (*cis*,*cis*,*trans*-diaminodichlorido-dihydroxido platinum(IV), Figure 1), shows increased inertness and therefore can be applied orally [10]. Oxoplatin was characterized by Chugaev and Khlopin for the first time in 1927 [11]. Oxoplatin was furthermore found to accumulate in tumors and metabolisis resulted in the formation of several complexes, amongst them cisplatin, pointing to a putative role of oxoplatin as a prodrug of cisplatin. In *in vitro* cytotoxicity tests employing a panel of 38 human cancer cell lines the IC_50_ values for oxoplatin proved to be 2.5-fold higher than those for cisplatin [10].

Survival of a fraction of tumor cells and development of chemoresistance may be influenced by an initial cellular stress response against the xenobiotics administered [12]. For example, the cisplatin-induced cellular stress response in NCI-H526 cells involved activation of p38α MAPK, whereas titanocene Y-triggered signaling affected c-Jun N-terminal kinase (JNK) [13]. Phosphorylation of adenosine monophosphate (AMP)-activated protein kinase α1 (AMPKα1), which promotes cell survival, was increased by both drugs [13]. The cisplatin-DNA adducts consist of interstrand and intrastrand DNA crosslinks, thus creating doublestrand breaks which in turn activate the cellular damage response. In general, DNA damage signaling involves activation of ATM and/or ATM- and Rad3-related (ATR), resulting in phosphorylation and stabilization of p53 [14,15]. Generally, p53 controls cell cycle checkpoints and induces growth arrest and either activates DNA repair or eliminates tumor cells which are beyond repair, when present as functional wildtype p53 [15]. Extracellular signal-regulated kinases (ERKs), JNK stress-activated protein kinase and p38α/β/γ MAPKs are activated by cisplatin and either promote survival or induce apoptotic cell death [15]. In the present study, the short-term effects of cisplatin and oxoplatin on phosphorylation of 46 sites of a total of 38 signaling proteins were compared in p53-wild-type NCI-H526 SCLC cells. To our knowledge, this is the first work comparing a platinum(II) drug and its putative platinum(IV) prodrug for their effects on a broader panel of the signal transduction phosphoproteins.

## 2. Results

### 2.1. Cisplatin- and Oxoplatin-Induced Alterations of the NCI-H526 Phosphome

**Figure 2:**
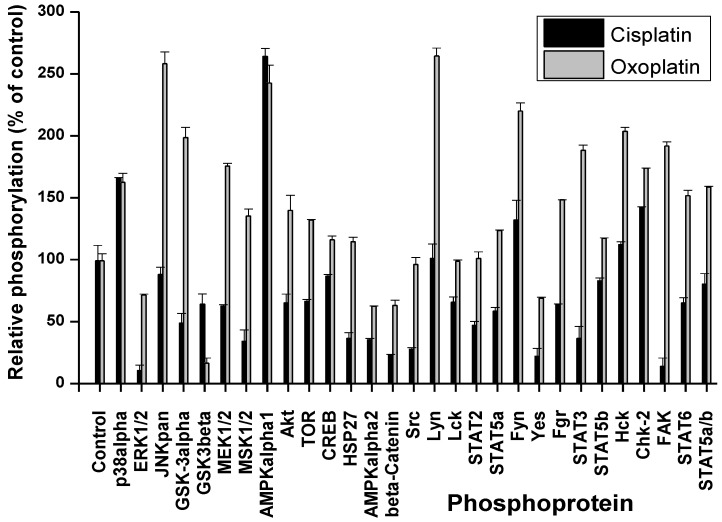
Comparison of the phosphoprotein response of NCI-H526 cells to short-term exposure to cisplatin and oxoplatin, respectively. Status of the phosphorylation of the proteins covered by array part A is presented as percentage of the untreated control cells (±SD; all differences in phosphorylation between oxoplatin and cisplatin exposure are significant with *p* < 0.049, except for p38α MAPK and AMPKα1).

**Figure 3:**
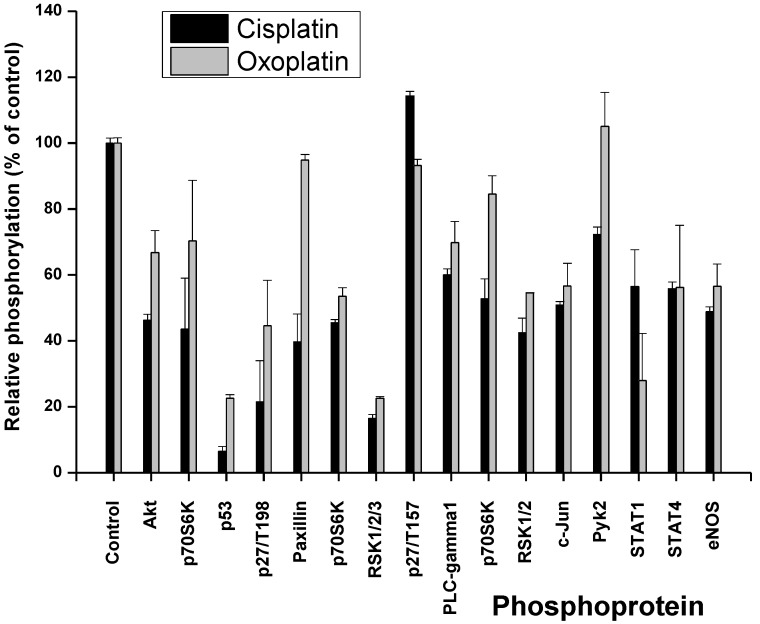
Comparison of the phosphoprotein response of NCI-H526 cells to short-term exposure to cisplatin and oxoplatin, respectively. Status of the phosphorylation of the proteins covered by array part B is presented as percentage of the untreated control cells (±SD; all differences between oxoplatin and cisplatin exposure are significant with *p* < 0.46, except for p70S6K, p27/T198, PLCγ1, c-JUN, STAT4 and eNOS).

Cells were treated for 6 h with 3 µM cisplatin or 4 µM oxoplatin in tissue culture medium, respectively. These concentrations correspond to the mean peak plasma concentrations of cisplatin in patients and the equipotent amount of oxoplatin, respectively [16]. Thereafter, cells were lysed and processed using the Profiler Human Phospho-Kinase Array Kit ARY003 (A and B panel of phosphoproteins; R&D Systems, Minneapolis, MN, USA) according to the manufacturer’s instructions. Changes in the phosphorylation of selected cellular proteins of NCI-H526 cells are shown in Figure 2 and Figure 3, relative to untreated control cells. 

Exposure of the cells to cisplatin resulted in increased phosphorylation of p38α MAPK, AMPKα1 and minor activation of the src kinases Fyn and HcK as well as Chk-2 for the A panel of phosphoproteins (Figure 2). Phosphorylation of all other proteins included in this assay were downregulated, with the single exception of Lyn. In contrast, treatment with oxoplatin provoked markedly increased phosphorylation of JNK, GSK-3, MEK1/2, MSK1/2, AMPKα1, Akt, TOR, Lyn, Fgr, HcK, STATs, Chk-2, and especially FAK (Figure 2). The remaining proteins showed unchanged or downregulated phosphorylation status. The second part of the assay (B) revealed downregulation of the phosphorylation of proteins in response to cisplatin, with single exception of p27/T157 (Figure 3). A similar downregulation in phosphorylation was found after exposure of the cells to oxoplatin, with exception of paxillin, p27/T157 and pyk2 which remained at control cell levels. Phosphorylation of p53 was reduced in response to both oxoplatin and cisplatin.

### 2.2. Effects of Short-Term Exposure of NCI-H526 Cells to Oxoplatin or Cisplatin on Cell Viability

**Figure 4:**
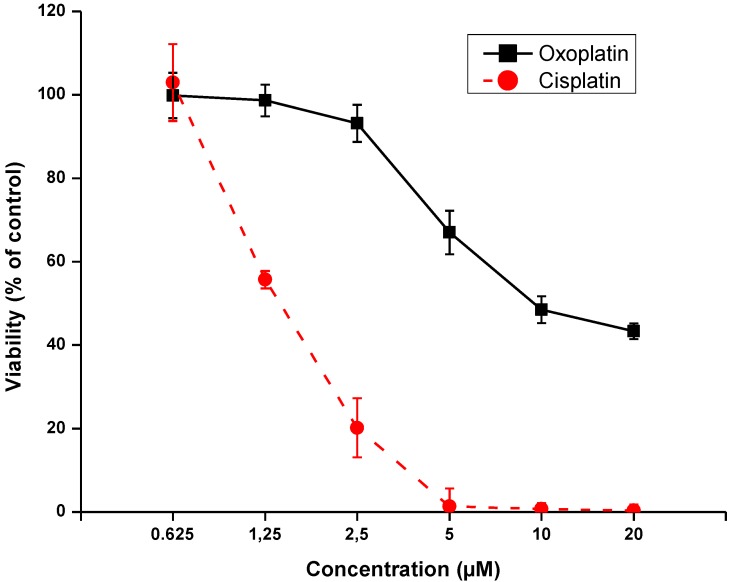
Viability of NCI-H526 cells exposed to either oxoplatin or cisplatin at six different drug concentrations for four hours and subsequently kept for four days in tissue culture prior to the MTT assay (all values significantly different with *p* < 0.001, except for the non-significant difference at the lowest drug concentrations used).

Aliquots of NCI-H526 cell suspensions were treated with 0.625–20 µM oxoplatin or cisplatin for four hours, respectively. Subsequently, cells were cultured for four days in fresh medium and checked for their viability (Figure 4). Results of the MTT assays revealed that up to 40% of the cells survived exposure to the highest concentration of oxoplatin, whereas, in case of cisplatin, tumor cells were not able to survive short-term exposure to 5–20 µM of this drug.

## 3. Discussion

In order to improve the clinical applicability platinum-based drugs and to reduce side effects and induction of drug resistance, as observed in the case of cisplatin, a host of different analogs were developed, with special consideration of an orally administrated platinum(IV) drug [4,5,9]. The most advanced kind of this chemotherapeutic, namely satraplatin, failed to gain approval, as well as a range of other platinum complexes. The reasons for these failures were manifold, comprising unfavorable pharmacokinetics, insufficient accumulation in tumor tissues, inefficient activation of prodrugs, diverse mechanisms of resistance and clinical tackling of different-to-treat cancer entities such as prostate cancer [4,5,7]. Resistance to platinum complexes is mainly attributed to decreased drug uptake, inactivation by binding to metallothioneins or glutathione, or increased repair or replicative bypass of drug-DNA adducts, respectively [14,15]. Cytotoxic platinum compounds, including cisplatin, are standard cancer chemotherapeutics and are also activators of stress-signaling pathways. The initial stress response of tumor cells to xenobiotics, including platinum-based drugs, is only partially clear and not considered as additional therapeutic target so far. 

We have investigated the alterations of the a panel of phosphoprotein kinases after short-term exposure of a he NCI-H526 SCLC cell line to cisplatin and a titanocene, respectively, and found p38α MAPK and AMPKα1 as essential components of adaption of cell signaling pathways in response to cisplatin [13]. In the present study, this work was extended to characterize the cellular response to the platinum(IV) drug oxoplatin in comparison to platinum(II) cisplatin. In a former study, oxoplatin was found to exhibited partial activity throughout a panel of cells derived from different tumor entities with IC_50 _values ranging from 0.6–120 μM, with a mean IC_50_ of 22.8 ± 17.4 μM for sensitive cell lines defined by an IC_50_ below 60 μM, comparing to a mean IC_50_ of 10.1 ± 9.1 μM for cisplatin for the same group of cell lines [10]. Platinum(IV) molecules are believed to represent relatively stable prodrugs that reach their target cells unaltered and bind to DNA and exert cytotoxic effects rapidly after reduction to the corresponding platinum(II) species [9]. Although oxoplatin is generally considered as prodrug of cisplatin, studies point to differences in intracellular effects in tumor cells through different activation pathways and intermediates [10,17]. 

As reported previously, short-term treatment of the NCI-H526 cells with cisplatin resulted in increased phosphorylation of p38α MAPK, AMPKα1 and minor activation of the src kinases Fyn and HcK, as well as Chk-2 for the first panel of phosphoproteins of the array [13]. Under this condition, phosphorylation of all other proteins included in this assay were downregulated, with the single exception of Lyn. In contrast, short-term exposure of the cells to oxoplatin provoked significantly increased phosphorylation of JNK, GSK-3, MEK1/2, MSK1/2, AMPKα1, Akt, TOR, Lyn, Fgr, HcK, STATs, Chk-2 and, especially, FAK in addition to the shared increase in phosphorylation of p38α MAPK and AMPKα1. For the NCI-H526 cell line, activation of p38α MAPK was previously shown to enhance cytotoxicity of cisplatin, whereas increased phosphorylation of AMPKα1 provided protection [13]. This corroborates previous reports, where AMPKα1 was demonstrated to represent an important factor for the sensitivity to cisplatin in colon and ovarian cancers, most likely by induction of autophagy [18].

Cisplatin was reported to stimulate JNK activity in SCLC cells. However, the cellular response to the less cytotoxic *trans*-isomer, transplatin, was rapid and transient, whereas JNK activation by cisplatin was delayed and sustained [19]. Activation of the JNKs by platinum compounds was demonstrated to control c-Jun-dependent transcriptional events that promoted protection of the cells. This situation may be similar here for the less active oxoplatin, inducing a rapid JNK response in contrast to cisplatin. Additionally, the host of phosphoproteins induced in response to oxoplatin seem to be involved in drug resistance mechanisms uniformly. Phosphorylation of GSK3 was found to be involved in cisplatin resistance in A549/DPP NSCLC and ovarian cancer cells [20,21]. In ovarian cancer cell lines, ERK2-mediated mitogen-activated protein kinase phosphatase-1 (MKP-1) phosphorylation was reported to be critical for cisplatin resistance [22,23]. Inhibition of ERK2 activity by the MEK1/2 inhibitor U0126 or by small interfering RNA silencing decreased MKP-1 induction, leading to an increase in cisplatin-induced cell death. MPK-1 transcription is also targeted by MSK1 and -2 and their downstream effectors cAMP-response element-binding protein (CREB)/ATF1 [24]. 

The phosphatidylinositol 3 kinase (PI3K) pathway is a key player in regulating cell proliferation, metabolic pathways, cell survival, and angiogenesis [25]. Thus, increases in the phosphorylation of Akt and TOR again contribute to increased survival following exposure to cytotoxic drugs [25]. This was confirmed in several tumor entities, such as lung and ovarian cancer and melanoma [26,27,28]. c-Src is a tyrosine kinase participating in control of tumor growth and dissemination which has been shown to impact the cellular response to cisplatin-induced DNA damages [29]. c-Src is frequently activated in NSCLC and transitional bladder cancer and administration of the Src inhibitor dasatinib abolished Src phosphorylation and sensitized lung and bladder cancer cells to cisplatin [29,30]. Activated Src phosphorylated the gap junction protein connexin 43, decreased gap junction communication, and increased cisplatin resistance in fibroblasts [31]. Similarly, other members of the src kinase family, such as Lyn, Fyn, Fgr and Hck, are likewise involved in cellular stress response and drug resistance [32,33].

Signal transducer and activator of transcription (STAT) comprises a total of six related transcription factors which are functional in inflammation, oncogenesis, suppression of cell death, proliferation and formation of metastases in malignant diseases [34]. STAT3 controls genes which promote survival/chemoresistance (survivin, bcl-xl, mcl-1, and others), proliferation (oncogenes and cell cycle kinases), and participate in invasion, such as metalloproteinases. Increased expression of constitutive STAT3 may suppress apoptotic cell death in inherently chemoresistant NSCLC cells [35]. The STAT3 pathway responded early to platinum drugs associated with cisplatin resistance in epithelial ovarian cancer [36,37]. Knock-down of STAT3 by specific siRNA restored cisplatin sensitivity of ovarian cancer cells [38,39,40,41]. Likewise, STAT5 can promote proliferation and inhibit apoptosis [42]. In head and neck cancer tumor patients, STAT6 expression is correlated with an improved clinical outcome for patients receiving platinum-based radiochemotherapy [43].

Chk1 and Chk2 are two checkpoint kinases that become phosphorylated by ATR kinase in response to cisplatin-triggered DNA damage [44]. Inhibition of Chk2 impairs cisplatin-induced p53 activation and apoptosis. The most divergent change in the phosphorylation pattern in response to the two platinum drugs affects FAK. In NSCLC cells, HGF reduced expression of apoptosis-inducing factor (AIF) and cisplatin sensitivity via HGF receptor (c-MET) and the downstream effector FAK [45,46]. OAW42-R ovarian carcinoma cells displayed a strong resistance to cisplatin-induced apoptosis in combination with a hyperphosphorylation of FAK [47]. Cell adhesion to fibronectin and phosphorylation of FAK, which are associated with alpha5beta1 integrin and involved in a cell survival signaling, were found to be increased in the cisplatin-resistant squamous cell carcinoma cells [48]. Thus, oxoplatin induced an extensive stress response, affecting a host of signaling pathways involved in drug resistance, contrary to the limited response observed for cisplatin. In contrast to cisplatin, paxillin and Pyk2 remained at control cell levels after oxoplatin treatment. Phosphorylation of paxillin via the Src pathway was responsible for cisplatin resistance of NSCLC cells [49]. Proline rich tyrosine kinase 2 (Pyk2) on promoting cisplatin resistance of HCC cells through preventing cell apoptosis, activation of AKT pathway and upregulation of drug resistant genes [50]. 

Our group has reported a similar cytotoxic response of NCI-H526 cells to oxoplatin and cisplatin, respectively, following a continuous exposure of the cells for 96 h [10]. However, short-term treatment resembles the *in vivo* exposure of the tumor cells to peak plasma concentrations and subsequent decline of the drug more closely. As demonstrated here, using short-term treatment of the tumor cells with oxoplatin resulted in a significant survival of a fraction of the tumor cells, in contrast to the situation observed for cisplatin. The previous results showed that long-term application of oxoplatin eventually overcomes the initial stress response, most likely by increasing conversion of this prodrug to cytotoxic metabolites. In general, reduction of platinum(IV) compounds may proceed in the bloodstream or intracellularly and is executed by reducing agents such as ascorbic acid, methionine, cysteine, glutathione, uric or lactic acid, and sulfhydryl-group containing proteins [51]. Thus, the cellular stress response may depend on the site of metabolization of a prodrug, generally becoming more intensely upon cellular internalization of the inactive precursor. In conclusion, the results obtained in this SCLC cell line using a platinum(IV) and a platinum(II) drug reveal that prodrugs may provoke a marked stress response until the active metabolite is generated and exerts cytotoxicity in tumor cells. 

## 4. Experimental

### 4.1. Chemicals

Unless otherwise noted, all chemicals were obtained from Sigma-Aldrich (St. Louis, MO, USA). Dulbecco’s phosphate buffered saline (PBS) was purchased from Gibco/Invitrogen (Carlsbad, CA, USA). Oxoplatin (*cis*, *cis*, *trans*-diaminodichlorido-dihydroxidoplatinum(IV)) was synthesized according to standard procedures by Chiracon (Luckenwalde, Germany) and kindly provided by IPSS (Berlin, Germany). Compounds were prepared as stock solutions of 2 mg/mL in either DMSO (oxoplatin) or in 0.9% NaCl solution (cisplatin), sterilized by filtration in case of cisplatin, and aliquots stored at −20 °C. 

### 4.2. Cell Culture

The NCI-H526 SCLC cell line was obtained from the American Type Culture Collection (Rockville, MD, USA). Cells were grown in suspension in RPMI-1640 bicarbonate medium (Seromed, Berlin, Germany), supplemented with 10% FBS (Seromed), 4 mM glutamine, and antibiotics (final concentrations: 50 U/mL of penicillin, 50 µg/mL of streptomycin, and 100 µg/mL neomycin; Sigma-Aldrich), and subcultivated twice a week. DNA profiling by short tandem repeat analysis of the NCI-H526 cells proved their identity to the American Type Culture Collection specifications, and the yeast p53 functional assay revealed expression of fully active p53 (functional assay of separated alleles in yeast FASAY; data not shown). 

### 4.3. Phosphokinase Array

Relative protein phosphorylation levels of 38 selected proteins were obtained by analysis of 46 specific phosphorylation sites using the Proteome Profiler Human Phospho-Kinase Array Kit ARY003 in duplicate according to the manufacturer’s instructions. Briefly, cells were rinsed with PBS, 1 × 10^7^ cells/mL lysis buffer were solubilized under permanent shaking at 4 °C for 30 min, and aliquots of the lysates were stored frozen at −80 °C. After blocking, membranes with spotted catcher antibodies were incubated with diluted cell lysates at 4 °C overnight. Thereafter, cocktails of biotinylated detection antibodies were added at room temperature for 2 h. Phosphorylated proteins were revealed using streptavidin-HRP/chemiluminescence substrate (SuperSignal West Pico, Thermo Fisher Scientific, Rockford, IL, USA) and autoradiography films (Amersham Hyperfilm ECL, GE Healthcare Life Sciences, Buckinghamshire, UK). The resulting spots were scanned and images were quantified using the ImageQuant TL v2005 software (Amersham Biosciences, Buckinghamshire, UK) and Microsoft Excel software (Microsoft, Redmond, WA, USA). 

### 4.4. Cytotoxicity Assay

Aliquots of 5 × 10^5^ NCI-H526 cells in 1 mL medium were treated for four hours with twofold dilutions of cisplatin or oxoplatin, respectively. At the end of incubation, the platinum compounds were removed by centrifugation and, thereafter, washed cells representing distinct platinum concentration steps were distributed into 96-well microtiter plates in quadruplicate (Greiner, Kremsmuenster, Austria). The plates were incubated under tissue culture conditions for four days and cell viability was measured using a modified MTT (3-(4,5-dimethylthiazol-2-yl)-2,5-diphenyltetrazolium bromide) assay (EZ4U, Biomedica, Vienna, Austria). Optical density was measured using a microplate reader at 450 nm with an empty well as reference. Values obtained from control wells containing cells and media alone were set to 100% proliferation. 

### 4.5. Statistics

Statistical analysis was performed using Student’s *t* test for normally distributed samples (* *p* < 0.05 was regarded as statistically significant). Values are shown as mean ± SD. 

## 5. Conclusions

In contrast to the platinum(II) compound cisplatin which induced a limited stress response in NCI-H526 SCLC cells, the platinum(IV) prodrug oxoplatin provoked a massive alteration of the phosphoprotein pattern, which is associated with stress response and increased short-term drug resistance. Thus, the initial exposure of cancer cells to a platinum-based prodrug with low inherent cytotoxic activity seem to trigger a broad cellular stress response that is expected to impair the effectiveness of the appearing active metabolite subsequently. Such an extended cellular stress response to a low active prodrug may have contributed, in conjunction with other limiting factors, to the failure of several platinum(IV) drugs to show sufficient clinical activity and represent a general model of chemoresistance to platinum-based prodrugs containing other moieties attached.
